# Collaborative improvement on acute opioid prescribing among diverse health systems

**DOI:** 10.1371/journal.pone.0270179

**Published:** 2022-06-23

**Authors:** Casey M. Clements, Kristine T. Hanson, Kathryn W. Zavaleta, Amber M. Stitz, Sean E. Clark, Randy R. Schwarz, Jessica R. Homan, Mark V. Larson, Elizabeth B. Habermann, Halena M. Gazelka, on behalf of the Mayo Clinic Care Network Acute Opioid Prescribing Collaborative

**Affiliations:** 1 Department of Emergency Medicine, Mayo Clinic, Rochester, MN, United States of America; 2 Department of Health Sciences Research, Mayo Clinic, Rochester, MN, United States of America; 3 Management, Engineering, and Consulting, Mayo Clinic, Rochester, MN, United States of America; 4 Department of Nursing, Mayo Clinic, Rochester, MN, United States of America; 5 Quality Academy, Mayo Clinic, Rochester, MN, United States of America; 6 Department of Provider Relations, Mayo Clinic, Rochester, MN, United States of America; 7 Division of Gastroenterology, Department of Medicine, Mayo Clinic, Rochester, MN, United States of America; 8 Department of Anesthesiology & Perioperative Medicine, Mayo Clinic, Rochester, MN, United States of America; University of Pretoria, SOUTH AFRICA

## Abstract

**Background:**

Despite broad awareness of the opioid epidemic and the understanding that patients require much fewer opioids than traditionally prescribed, improvement efforts to decrease prescribing have only produced modest advances in recent years.

**Methods and findings:**

By using a collaborative model for shared expertise and accountability, nine diverse health care systems completed quality improvement projects together over the course of one year to reduce opioid prescriptions for acute pain. The collaborative approach was flexible to each individual system’s goals, and seven of the nine participant institutions definitively achieved their desired results.

**Conclusions:**

This report demonstrates the utility of a collaborative model of improvement to bring about real change in opioid prescribing practices and may inform quality improvement efforts at other institutions.

## Introduction

The opioid epidemic continues across the United States, accounting for 47,600 overdose deaths (67.8% of all overdose deaths) in 2017 [1]. While illicit drugs, such as heroin, account for many deaths, available data show that up to 82.6% of opioid drug users start by using a prescription medication [2], placing the epidemic squarely within the scope of healthcare industries. In addition to the staggering mortality, opioid use disorder causes significant morbidity to patients as well as financial impact in both healthcare expense and productivity losses accounting for a combined annual cost of $504 billion to American society [3].

Opioid medications were historically thought to be low risk for misuse or addiction and were marketed as such to prescribers for many years [4, 5]. However, more recent and robust evidence shows that patients, including those who are opioid naïve, are at significant risk of developing long term opioid use after even a single prescription [6–8]. This risk for patients developing long term use varies based on the clinical setting in which medications are prescribed and the indication for the use of the medication, where the risk of developing long-term use ranges from 2% to as high as 30% [9–11], with about 6% of previously opioid naïve surgical patients developing chronic opioid use post-operatively [6, 7].

Despite significant attention to opioid prescribing, including legislation in several states, most systems have only shown modest decreases in opioid prescribing [11, 12]. Interestingly, patients tend to need significantly fewer doses of opioid medications than we often prescribe for their acute pain, suggesting that prescribing habits are driven by historical practices and not patients’ use patterns [13–17]. Such excess prescribing increases availability of medications for diversion and/or misuse. Indeed, most drug diversion and misuse is of lawfully obtained medications, and as many as 25% of opioids dispensed in the United States are used nonmedically [18]. Indeed, the amount of medication in a prescription is thought to be the greatest risk factor for opioid prescription misuse [19]. This suggests an opportunity for improvement, whereby bringing prescribing practices in line with patient needs can decrease the risk of misuse while adequately addressing patients’ pain management.

Ever since the Institute of Medicine’s publication of *To Err is Human* (2000) and *Crossing the Quality Chasm* (2001), healthcare systems have endeavored to bring quality improvement methodologies from other industries to healthcare settings. The DMAIC (Define, Measure, Analyze, Improve and Control) method, popularized by Six Sigma projects, is frequently used to achieve goals in individual healthcare units, hospitals/clinics, and health systems. Moving beyond individual disciplines and institutions, The Institute for Healthcare Improvement (IHI) developed the “Breakthrough Series,” a collaborative learning model for addressing difficult problems in healthcare. Early on, this model successfully improved cesarean section rates, waiting times in clinics, and asthma care utilization at several institutions [20]. The Mayo Clinic Care Network (MCCN) was launched in 2011 as a group of like-minded organizations with the goal of optimizing patient-centered care without respect to geography. The MCCN now has 45 members in 9 countries, representing 96 hospitals, over 18,000 physicians, and has the potential to reach more than 17 million patients. The MCCN has successfully used a shared learning and expertise model for collaborative improvements among multiple health systems in the past for improvements such as enhanced recovery following colon and rectal surgery [21, 22] and others as well. MCCN members are invited to participate in such collaboratives, including the one we report here: improving acute opioid prescribing. We aim to describe our experience with this collaborative in order to inform future initiatives aimed at reducing opioid prescribing and demonstrate the utility of a collaborative in these efforts.

## Methods

MCCN members were invited to participate in a quality improvement collaborative to improve opioid prescribing. Over the course of a year, Mayo Clinic participants facilitated goal setting and implementation of quality improvement projects at each institution to reduce opioid prescribing. Each member organization conducted an improvement project over 12 months with monthly coaching calls including subject matter experts and quality improvement specialists from the collaborative. Participating organizations met both face to face and via web meetings to discuss their progress and challenges. Here we detail the actions that took place during each stage of this collaborative work.

### Pre face-to-face preparation

MCCN members were invited to an initial informational webinar regarding the Acute Opioid Prescribing Reduction Collaborative. This webinar was designed to allow members to hear an overview of the proposed program, review project goals, gauge interest in the topic and secure commitment from interested member organizations. Mayo also shared successes in reducing acute opioid prescribing. Care network members were then given a deadline to express their interest in participating in this collaborative. Members who expressed interest were then invited to a second kick-off webinar and subsequent calls.

During the second webinar, current state mapping and baseline data requirements were discussed. As quality improvement of existing practices, this work was deemed exempt from IRB review. Data were compiled by the institutions and submitted anonymized to the collaborative team. Discussion also took place to help members understand which staff roles from their organizations should travel to Rochester, MN for the initial face-to-face meeting.

A final pre-meeting call was scheduled before the face-to-face event. During the pre-meeting call, members were encouraged to ask any questions they had about the collaborative prior to the face-to-face meeting. Many of the questions asked during this call were centered on the baseline data that was requested and selecting the most appropriate team members that should travel to Rochester for the initial face-to-face meeting.

### Collaborative participants

**Fig 1:**
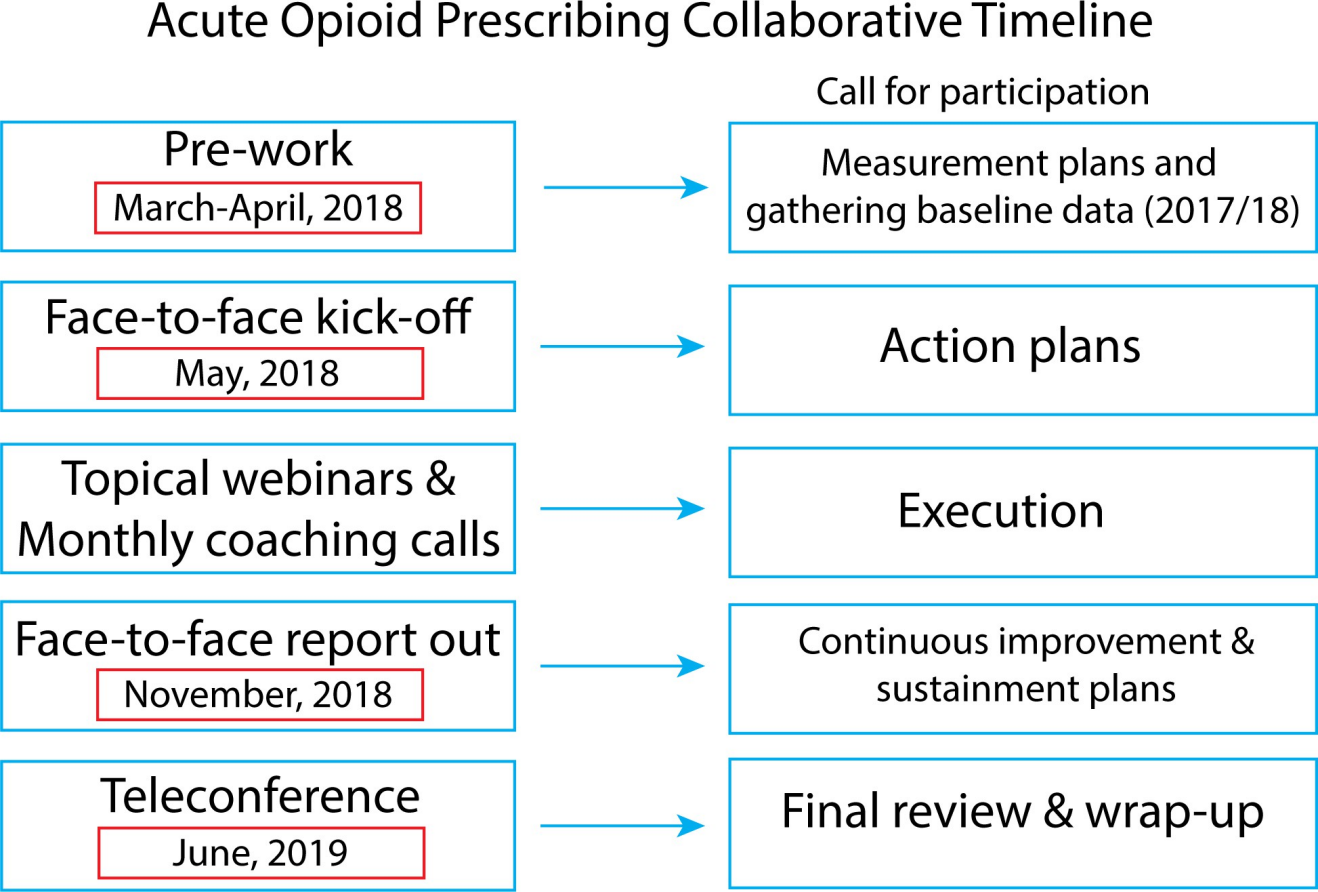
Project timeline.

Nine MCCN health systems from across the United States chose to participate in the acute opioid prescribing reduction collaborative. These nine systems represented both single and multihospital organizations serving patients in urban, suburban, and rural settings representing a combined 4,262 physicians serving a population of 2,876,850 patients (Table 1). The collaborative took place over 12 months with frequent and significant interaction between participants and Mayo Clinic staff (Fig 1, details in S2 Table).

**Table 1:** Participating institutions.

Care Network Member	# of physicians^1^	# of patients^2^	Locations	Community Served	Improvement goal	Goal achieved	Initial reduction^3^	Final Follow-up
A.	302	203,850	Single site	Rural	5% OME reduction for post-operative knee/hip replacement patients	Yes	67%	67%
B.	531	358,425	Multisite	Urban	10–20% OME reduction in several post-operative patients	Yes	27%	N/A
C.	412	278,100	Single Site	Rural	OME reduction for post-operative knee/hip replacement patients to <400 OME	Yes	29%	N/A
D.	1,135	766,125	Multisite	Urban, Suburban & Rural	Reduce overall inpatient use	Yes	9% inpatient, 17% discharge	N/A^4^
E.	805	543,375	Single Site	Urban	Reduce ED traumatic back pain prescriptions 50%	Yes	55%	69%
F.	135	91,125	Single Site	Rural	20% reduction in variability of prescribing by procedure	Unknown		
G.	658	444,150	Single Site	Urban	50% reduction in orthopedic post-operative prescribing	No	-8%	-12%
H.	177	119,475	Multisite	Rural	Reduce post-operative prescribing & lead regional efforts with professional and government organizations	Yes	6%	46%
I.	107	72,225	Single Site	Rural	Improve orthopedic prescribing, decreasing variability	Yes	33%	7%
Total	4,262	2,876,850						

^1^ Number of physicians who have access to MCCN tools/services

^2^ Number of patients served by those physicians

^3^ See Table 2 for data and statistical analysis

^4^ This institution focused on inpatient use and did not measure this at the final data entry, but did provide outpatient prescribing information as shown in Table 2

**Table 2:** Opioid prescribing improvement data.

	Baseline			Initial Follow-up				Final Follow-up			
**Care Network Member**	Dates^1^	N (%) opioids^2^	OME Median (IQR)	Dates^1^	N (%) opioids^2^	OME Median (IQR)	p-value^3^	Dates^1^	N (%) opioids^2^	OME Median (IQR)	p-value^3^
**Acute Opioid Prescribing at Discharge for Patients Undergoing a Surgical Procedure**											
A.	1/1/2017-12/29/2017	360 / 360 (100.0%)	690 (465, 900)	8/16/2018-10/2/2018	27 / 27 (100.0%)	225 (225, 225)	<0.001	1/10/2019-3/28/2019	32 / 32 (100.0%)	225 (225, 300)	<0.001
B.	3/4/2017-2/2/2018	27 / 30 (90.0%)	375 (210, 472.5)	6/5/2018-9/3/2018	34 / 34 (100%)	275 (150, 500)	0.66	*Not available*			
C.	4/6/2017-4/4/2018	768 / 774 (99.2%)	450 (375, 672)	8/3/2018-10/1/2018	119 / 119 (100.0%)	320 (225, 400)	<0.001	*Not available*			
D.	1/5/2017-1/8/2018	1112 / 1112 (100.0%)	300 (165, 600)	6/1/2018-10/23/2018	351 / 351 (100%)	300 (150, 450)	0.16	10/24/2018-6/23/2019	6676 / 6682 (99.9%)	225 (150, 300)	<0.001
F.	1/1/2018-4/29/2018	32 / 65 (49.2%)	0 (0, 225)	7/7/2018-9/3/2018	19 / 34 (55.9%)	0 (0, 225)	0.98	3/6/2019-5/2/2019	18 / 46 (39.1%)	0 (0, 225)	0.39
G.	11/2/2017-11/17/2017	29 / 30 (96.7%)	600 (500, 800)	8/7/2018-8/21/2018	30 / 30 (100.0%)	650 (350, 700)	0.11	12/4/2018-12/31/2018	28 / 30 (93.3%)	675 (500, 700)	0.17
H.	1/4/2018-3/31/2018	34 / 42 (81.0%)	375 (200, 450)	6/7/2018-9/20/2018	31 / 33 (93.9%)	352.5 (200, 450)	0.59	1/1/2019-4/11/2019	34 / 39 (87.2%)	200 (175, 315)	0.003
I.	1/6/2017-3/30/2017	27 / 31 (87.1%)	150 (100, 225)	6/2/2018-6/24/2018	9 /12 (75.0%)	100 (0, 150)	0.03	12/2/2018-3/29/2019	25 /32 (78.1%)	140 (75, 225)	0.22
All	1/1/2017-4/29/2018	2389 / 2444 (97.7%)	400 (240, 672)	6/1/2018-10/23/2018	620 / 640 (96.9)%	300 (165, 450)	<0.001	10/24/2018-6/23/2019	6813 / 6861 (99.3%)	225 (150, 300)	<0.001
**Acute Opioid Prescribing in Emergency Medicine**											
E.	1/2/2017-6/30/2017	12 / 30 (40.0%)	0 (0, 67.5)	9/16/2018-10/15/2018	9 / 51 (17.6%)	0 (0, 0)	0.01	1/1/2019-3/29/2019	5 / 40 (12.5%)	0 (0, 0)	0.008

Abbreviations: OME, Oral Morphine Equivalents; IQR, Interquartile Range

^1^ Dates of discharge

^2^ Number of patients prescribed an opioid at discharge / Number of cases reviewed

^3^ Distribution of OME compared to baseline

### First face-to-face meeting

The collaborative began with a face-to-face meeting, which took place in Rochester, MN over a period of one- and one-half days. The MCCN teams were encouraged to bring a physician leader/champion, nursing leaders, administrative partners, and other key stakeholders such as pharmacists and advanced practice providers.

The evening before the face-to-face meeting a dinner reception was held with care network members and course faculty. This reception set the stage for collaboration during the event and allowed participants to network with each other and with the Mayo Clinic’s course directors and subject matter experts.

On day one of the face-to-face meeting, participants were welcomed by the physician leaders and course directors. During the first day, critical didactic content was delivered by key subject matter experts. Following those presentations, quality improvement strategies were discussed, and then MCCN member teams were given dedicated time to begin to plan their initial action steps.

On day two, we incorporated the use of a panel discussion to allow informal time for questions and answers. This panel was convened of various subject matter experts (SMEs) from around Mayo Clinic Rochester. Their topic was: “A Multidisciplinary Look at Opioid Management.” These panel members spent time informally answering questions from members on acute opioid prescribing best practices.

Also on day two, a member roundtable was convened. During this roundtable, care network members had a chance to report out on their current state for acute prescribing and what gaps were identified during the program, setting early goals for their improvements. The roundtable also provided an opportunity for the assembled teams to map out the first steps they were planning on taking after returning back to their organizations. The meeting concluded with the review of the timetable for the collaborative, which included monthly touch-point tele-conference calls, and the date and plan for the final report out face-to-face meeting in 6 months.

### Coaching calls

In the intervening 6 months between the two face-to-face meetings, touch point calls between Mayo Clinic and the care network members were held monthly with each health system to discuss how organizations were progressing on their project, to allow the Mayo Clinic team to offer assistance and insight to address quality and support needs, and to answer any questions or address organizational or clinical barriers that may have arisen since the face-to-face meeting. These meetings also served as a regular checkpoint to ensure projects were staying on track.

### Webinars

In addition to the monthly coaching calls, a series of three webinars were held between the first face-to-face event and the report out event. These webinars were designed around topics that our members requested more time and discussion on. The timing and details on these events are further detailed in S2 Table. The webinar topics were chosen to address needs of the collaborative and to address challenges that were commonly experienced by the members. The topics were as follows:

- Driver Diagrams and Quality Improvement Resources
- Opioid Disposal
- Alternative Pain Management

### Reporting out—One day face-to-face event

The report out occurred as a one-day face-to-face event six months after the initial face-to-face meeting. During this report out, each team presented a templated slide presentation and shared the following:

- Elevator Speech to Leadership
- AIM Statement(s)
- Where are we today?
- Where do we still need help?
- What went well?
- Update on key common metrics

### Follow-up teleconference

A final report out occurred as a teleconference one year after the initial face-to-face meeting. Teams shared updates to the information shared at the 6-month report-out including:

- Successes, share 2–3 key accomplishments
- Describe work yet to be done to resolve other barriers that may thwart safe prescribing practices
- Plans for sustainment including important next steps
- Actionable metrics; describe your plan to use actionable metrics to monitor progress.
- Lessons learned
- Diffusion plans; actions taken or in progress to spread best practices across your organization

### Institutional project design

During the first face-to-face meeting, each participating team identified their goal and target population for their opioid reduction efforts. Each institution set their own goals, most of which were quantitative improvements in outpatient prescribing, although some chose to focus on qualitative improvements without a quantitative target. Of nine participating organizations, one elected to focus on emergency department (ED) based prescribing, focusing on prescribing for patients with the chief complaint of back pain. The remaining eight members focused on reducing postoperative opioid prescribing following a variety of surgical procedures, including orthopedic, spinal, vascular, gynecologic, gastrointestinal, colorectal, and hepatobiliary procedures. Most teams chose to focus on one or two patient populations (eg, joint revision and hysterectomy), while some expanded their goals across many surgical specialties (Table 1). The availability of published evidence on post-surgical prescribing practices motivated many of the organizations to pursue reductions in the amount of opioids prescribed, while other organizations focused on variability that they saw in their prescribing data, and others were motivated by perceived gaps in the current practice.

Participating teams employed a variety of interventions to achieve their aims. Interventions included implementing standard prescribing guidelines, protocols, and order sets; developing patient education materials focused on patients both at admission to set expectations and at discharge to provide support in home management and disposal; implementing post-discharge follow-up phone calls by care team members; and carrying out community education sessions and drug take-back events. Interventions focused on engaging leadership and staff included identifying organizational champions, developing multidisciplinary teams to lead prescribing change efforts, and disseminating comprehensive education content for providers, advanced practice leaders, pharmacists, nursing, and organizational leadership. Finally, interventions focused on information technology (IT) and the electronic health record (EHR) included standardizing electronic ordering processes, deploying IT and EHR builds for change requirements, and establishing data management and monitoring plans. As healthcare systems are complex, institutions employed a variety of methods in combination based on the needs of their individual cultures and goals. These tools were provided to them and assisted throughout the collaborative timeframe (Table 3).

**Table 3:** Quality improvement interventions.

Project Aims	Targeted Intervention
Reduce total OME inpatient setting	• Implement standard prescribing guidelines and protocols
	• Develop patient Education across the continuum of care:
	o Pre-operative education to establish pain expectations
	o Standard pain and opioid written or media material
	• Standardize surgical and anesthesia clinical management
	• Standardize inpatient order sets
Reduce OME at discharge from hospital	• Implement standardized opioid discharge prescribing guidelines and best practice standards
	• Standardize discharge order sets
	• Develop patient education material focused on home management and disposal
Reduce OME outpatient/ED prescriptions (population based)	• Developed and implement standard prescribing guidelines and protocols
	• Provide patient education on admission for establishing expectations
	• Standardized population based, emergency care order sets
Reduce prescribing variation and improve global prescribing practices	• Identify and engage organizational champions
	• Establish a dedicated multidisciplinary leadership team to lead prescribing change efforts (e.g. Opioid Care Transformation Team or Opioid Steering Committee)
	• Evaluate baseline data metrics
	• Establish data management and monitoring plans (e.g. Tableau dashboard)
	• Develop usable and accessible prescribing toolkits and resources for providers and allied health staff
	• Develop and disseminate comprehensive education programs and content for providers, advanced practice providers, pharmacists, nursing, and organizational leadership
	• Assess informatics (IT) and Electronic Health Record (EHR) functionality to facilitate work flows and prescribing compliance
	• Deploy IT and EHR builds for change requirements
	• Standardize electronic ordering processes (i.e. order sets, smart sets, etc.)
Improve outpatient drug management and safety	• Community education and information sessions/events
	• Drug Take-back events
	• Implement post-discharge patient follow-up phone calls with care team member

### Data measurement

In order to assess baseline prescribing and improvement over the time period between the face-to-face meetings, Mayo developed a data collection form to capture opioid prescribing at each site at three time points: baseline (prior to the first face-to-face), interim (prior to the face-to-face report out), and follow-up teleconference (at 1 year). The collection form captured opioid prescribing at the patient level and included fields for inpatient vs outpatient, admission and discharge dates, procedure code (CPT or ICD-10), patient demographics, discharge physician and surgeon name and specialty, and details about each opioid prescription (medication generic and brand names, prescribing physician role and specialty, order date, quantity and units, strength and units, and timing of the prescription [e.g., ordered at discharge vs subsequent prescription]). Each site completed data abstraction for their target population at each of the three assessment time points and sent them to Mayo for analysis. The baseline data time frame preceded the improvement collaborative and included patients from 2017 and early 2018. A single contact at Mayo communicated between the data analyst and each site regarding questions about data inconsistencies that were found during data analysis.

### Data analysis

Prescriptions were converted to oral morphine milligram equivalents (OME), and analysis was limited to discharge prescriptions. For each site, patients prescribed an opioid at discharge was described as N (%), and total OME was described as median (interquartile range). Patients with missing or incongruent data for quantity or quantity units on any prescription were not included in calculations of OME.

Wilcoxon rank-sum tests compared OME at the interim follow-up and final follow-up to OME at baseline within each site as well as combined across the eight sites focusing on postoperative prescribing. All member sites participated in data collection at the baseline and interim follow-up time points, but due to resourcing and other local institutional constraints, only seven out of the nine sites were able to participate in data collection at the final follow-up time point.

## Results

### Baseline pre-intervention measurement

All pre-intervention data included at least 30 patients per system, with some systems including many more given differences in the size of the patient population as well as limitations within systems’ individual electronic health record and data structure. All post-operative baseline data were from patients discharged between January 2017 and April 2018. Among members focusing on postoperative prescribing (systems A through I), the median amount of opioids prescribed for post-operative patients was 400 OME, with an interquartile range (IQR) of 260–672 (Table 2).

### Post-intervention measurement

Data collected as the interventions were occurring were presented to the group as interim results at the second face-to-face gathering at the six-month mark. These data included patients discharged between June 2018 and October 2018. Within that time frame, four participant organizations had already achieved statistically significant reductions in discharge opioid OME (Table 2). Among the eight organizations focusing on postoperative prescribing, OME significantly decreased to median 300 (IQR 165–450) compared to baseline prescribing, p<0.001.

A final data collection was solicited at one year and included patients discharged between October 2018 and June 2019. Seven of the member organizations submitted the final dataset. Six of the participating organizations had significant reductions in the amount of opioids prescribed. Healthsystems A, C, D, H, and E had sustained decreases and healthsystem I had an initial decrease, though their prescribing increased slightly at the end of the study period. Seven of the nine participating health systems definitively met their predetermined, individual objectives. Among the six organizations focusing on postoperative prescribing (systems A through I) who submitted final data, OME significantly decreased to median 225 (IQR 150, 300) compared to baseline, p<0.001.

### Feedback and evaluation

During the collaborative, feedback was solicited from participating teams for what worked well and what needed improvement in the collaborative process. 21 leaders responded, representing all but one of the member organizations. All but two participants rated the collaborative overall as “excellent,” with one person saying “above average” and one “needs improvement.” Strong points included the curriculum and presentations that were described as “helpful,” “incorporating evidence,” and “free of commercial bias.” Mayo Clinic staff support for the teams was also very highly rated. Opportunities for improvement in future work included expanding inclusion of other non-opioid, non-pharmacologic, and multimodal therapy alternatives as well as even more time for various member organizations to interact and learn from one another.

## Discussion

This collaborative model of shared expertise and learning between institutions was successful at helping individual health systems complete concrete objectives, leading to reductions in opioid prescribing more accurately mirroring patient need. This brought about change for individual patients from projects supported on a national scale. Importantly, these interventions are customizable, as they are built on each institution’s goals and culture, portable, based on external collaboration and input, and also scalable, given similarities in post-operative and emergency care across hospitals.

Lessons learned from this collaborative may inform other health care administrators and quality improvement professionals on the utility of a collaborative in reducing opioid prescribing. Using a collaborative approach in these efforts was demonstrably successful; the Mayo Clinic Care Network is a collective entity of like healthcare organizations which benefit from a shared experience, and working together to identify and employ strategies yielded a greater impact on a higher number of patients. Furthermore, our efforts focused on clinically-based evidence, and interventions were built upon the demonstrated success of strategies previously implemented at Mayo Clinic. The varied interventions employed by the participating institutions may inform future efforts elsewhere.

This collaborative was successful due to several factors, but there are two that we feel should be emphasized. The first factor is the reliance on shared expertise and accountability. Between organizations, quality improvement experts partnered with bedside clinical staff with shared understanding of the needs of the clinical practice. This brings the expertise needed for implementing change successfully. Beyond that, however, collaborative participants held one another accountable both within organizations’ internal teams, as well as with Mayo Clinic staff who followed up with the teams on regular coaching calls and with data submission dates expected. Second, these projects were well supported by health systems’ leadership for both prioritization and resources. Support was ‘top down’ since the health systems’ leaders chose the collaborative teams, and asked them to participate in this project. Such endorsement was key in making changes, some of which required information technology support for dashboards or clinical decision support, acquiring funding, or staff time for training and participation. This level of support, or internal backing of the teams, was key to their successes.

Of the participant organizations, one healthsystem (I) made improvements and by the end of the collaborative had lost some of the gains they noticed initially. While the reasons for this are unclear in this particular circumstance, it raises the issue of sustainability. Control plans were presented and discussed throughout the collaborative to help foster long term improvements. There are likely also societal contributions to sustainability for opioid prescribing reductions since the opioid epidemic in the United States is well known at this point. Additionally, the focus of legislation on prescribing guidelines and monitoring initiatives in many states could help continue to prioritize these improvements.

Making improvements in opioid prescribing is challenging, and many factors affect prescribing that may confound our results. Certainly, the opioid epidemic has received a lot of attention during recent years and there may be a Hawthorne effect that could either bolster our efforts at decreasing prescribing or independently be influencing prescribers to decrease their opioid prescribing. Available evidence for this is unclear. Some data say that opioid prescribing is decreasing [12, 23], while other research has shown little to no effect in recent years [11].

Excellent pain control is needed when patients are suffering. Indeed, alleviation of that suffering is a core value of the field of medicine. Opioid medications are still often needed for treatment of the most severe pain. The strategies employed by this collaborative were to “right size” prescriptions where opioids were needed and not to prescribe opioids if they were not needed. In clinical situations where opioids were not thought to be needed, alternative pain control still needs to be provided whether that is through medications, procedures, or complementary therapy. The collaborative dedicated a webinar to alternative treatment options, but was not prescriptive in how to employ opioid alternatives. This is because interventions were intended to be pragmatic and customizable to both the health systems and patient populations targeted. While some health systems chose to focus on postoperative pain from significant procedures, others looked at lower acuity complaints such as idiopathic back pain. Clearly, a one-size-fits-all approach would not be possible for those very different populations. However, any group looking to shift therapy away from opioids should proactively consider alternative pain treatments as a key counterbalance measure to any opioid reduction quality improvement.

State legislation on opioid prescribing, particularly in those states pursuing prescribing limits, likely confounds our results somewhat. These legislative limits come in one of two general varieties: those that limit ‘days’ of medication prescribed, and those that limit amount by OME. Of these two general options, those that limit to ‘days’ of medication prescribed would complicate our results less so than those limiting OME. This is because of both how patients take a given medication and how prescribers write a prescription. Opioid medications are not intended to be taken all day, every day, but only when needed, so a supply measured in ‘days’ is significantly higher than many of our projects’ goals.

Several barriers are perceived in improving opioid prescribing. Many prescribers remain concerned that there may be decreased patient satisfaction when the amount of opioids prescribed is decreased. However, the literature available around this issue has not yet found that to be true [24]. Minimizing clerical burden is a top priority to combat burnout in medicine and additional requests on providers are to be actively avoided [25]. Previous work on post-operative prescribing reductions have not increased the rate of refill requests [17, 26], and post-visit phone calls from one of the collaborative members (Health System A) confirmed that patients indeed used less medication than prescribed. This would suggest that opioid reducing interventions were associated with good pain control following knee and hip replacement surgery, the chosen intervention population for health system A. However, none of the participant institutions directly measured patient reported pain control, which is a potential limitation of our study. As with any major change in clinical practice, ‘buy-in’ at all levels of care delivery is a concern for change leaders. This concern was expressed by all collaborative participants during the first face-to-face session. At the completion of the collaborative, six of the nine health systems specifically listed ‘buy-in’ and ‘engagement’ as something that “went well” for their projects, indicating that staff and leadership are likely more engaged with opioid improvement efforts than is perceived.

Our experiences presented here demonstrate the continuing utility of the IHI collaborative learning model approach in working towards the optimization of acute opioid prescribing across a range of settings and states with varying laws and restrictions. We learned several important lessons that will inform future work. First, acknowledging that there are significant, intrinsic differences between health systems is necessary. Goals of individual health care organizations vary, and hospitals and clinics are subject to different facilitators and challenges based on geographic and economic pressures within an area and health care market. In many ways, a smaller community setting may facilitate implementing change when systems are in a limited market; however, such places may not have the resources to bring improvement efforts to bear that larger institutions may have. Next, while much of this work is reproducible, the specific expertise and passion of individuals within any collaborative model cannot be fully replicated based on individuals’ strengths and talents. This collaborative is large enough that gaps are likely minimized. However, in order to repeat this process, attention to such talents or maintaining the critical mass of a collaborative may be needed to achieve these results. Lastly, the ability to scale and diffuse interventions is paramount in any challenging improvement development.

As the opioid epidemic continues, it remains paramount that health care institutions implement efforts to reduce levels of and variation in opioid prescribing. Our experiences demonstrate that the strengths of a collaborative representing diverse clinical practices are the breadth of expertise and varied solutions brought to bear, which may result in demonstrable impact to patient care and outcomes.

## Supporting information

S1 Table Acute opioid prescribing collaborative leadership teams. (DOCX)

S2 Table Opioid collaborative timeline–detailed. (DOCX)
