# Supplementary material for: Collaborative improvement on acute opioid prescribing among diverse health systems
Source: PLoS One. 2022 Jun 23;17(6):e0270179. doi: 10.1371/journal.pone.0270179 (PMC9223335; doi:10.1371/journal.pone.0270179)
Supplement: S1 Table — (DOCX) [file pone.0270179.s001.docx]

**S1 Table. Acute Opioid Prescribing Collaborative Leadership Teams.**

| **Mayo Clinic Care Network Member Organization** | **Key Staff** | **Role** |
| --- | --- | --- |
| Altru Health System, Grand Forks, ND | Ben Roth, MD | Family Medicine |
|  | Meghan Compton | Chief Legal Officer |
|  | Lynnette Huot | Director Quality & Safety |
|  | Erin Navarro | Manager Retail Pharmacy Services |
|  | Vinita Parikh, MD | Interventional Pain |
|  | Eric Lunn, MD | President |
|  | Lisa Johnson, RN | Director Primary Care |
| Beacon Health System, South Bend, IN | Deb Thompson | Executive Director Surgery, Trauma and Pulmonary Service |
|  | Melissa Demetriou MSN, RN, FNP-C | Nurse Practitioner |
|  | Mark Thompson, MD | Surgeon |
|  | Jim Galasso | Medication Safety Officer |
|  | Abhi Das, MD | Medical Director, Pain Management |
|  | Kashif Shaikh | Neurosurgeon |
|  | Don Jansen, MD | Vice President of Medical Affairs |
| Billings Clinic, Billings, MT | Dania Block, MSN, RN | Director, Medical Surgical Services |
|  | Kyle Townsend, PharmD | Director, Pharmacy Services |
|  | Erik Wood | Vice President, Ancillary Services |
|  | Bryon Hobby, MD | Orthopedic Physician |
| INTEGRIS Health, Oklahoma City, OK | Tommy Ibrahim, MD | Chief Medical Officer |
|  | Michael Thompson | Administrative Director Clinical Optimization |
|  | Jennifer Maune, Pharm.D., BCPS | Pharmacy Manager |
|  | Kerri Bayer, RN | Vice President |
|  | Benjamin Mansalis, MD | CMIO |
|  | Stewart Smith, MD | Neurosurgeon |
| Lakeland Regional Health, Lakeland, FL | Lisa Hartsfield RN, BSN | Clinical Systems Analyst |
|  | Maureen Leckie, MSN | Associate Vice President Clinical Operations |
|  | Nate Stephens, DO | ER Physician |
| Parrish Medical Center, Titusville, FL | Yashira Pabon-Padin, PharmD, BCPS | Clinical Pharmacist |
|  | Amy Jarrett, RN | Clinical Coordinator |
|  | Greg Cuculino, MD | Chair of Emergency Medicine |
|  | Alex Gutierrez, MSN, RN | Director, Med/Surg Nursing Division |
|  | Melinda Hodges RN, BSN | Nurse Manager Women's Services |
| Sparrow Health System, Lansing, MI | Kristin Gaumer, DO | Physician |
|  | Afrina Aziz, MD | Hospitalist |
|  | Ashley Meyers, BSN, RN-BC, PCCN-K | Nurse Educator |
|  | Mike Zaroukian, MD | Vice President & Chief Medical Information Officer |
|  | Parut Bhimalli, MD, FABPM | Medical Director of Pain Clinic |
|  | Lee King, PharmD | Medication Safety Officer |
|  | Anna Melville | Director, Population Health |
|  | Brian McCardel | Orthopaedic Surgeon |
| Unity Health, Searcy, AR | Justin Piker, Pharm.D., BCPS | Clinical Coordinator |
|  | Suporn Sukpraprut-Braaten, Ph.D. | Graduate Medical Education Research Director |
|  | Stacy Zimmerman, MD, FACP, FAAP | Internal Medicine Program Director |
|  | Justin O. Franz, MD | Orthopaedic Surgeon |
|  | Clark Osborne | Program Development and EMR Application Manager |
| Yuma Regional Medical Center, Yuma, AZ | Nicole Benvenuta, RN | Quality Analyst Coordinator |
|  | Monika Sanchez, RN, MSN | Director of Tower 4 Post Surgical Care Unit |
|  | Kwasi Wilson, M.D. | Medical Director |
