# Supplementary material for: Collaborative improvement on acute opioid prescribing among diverse health systems
Source: PLoS One. 2022 Jun 23;17(6):e0270179. doi: 10.1371/journal.pone.0270179 (PMC9223335; doi:10.1371/journal.pone.0270179)
Supplement: S2 Table — (DOCX) [file pone.0270179.s002.docx]

**S2 Table. Opioid Collaborative Timeline - Detailed.**

| **Phase** | **Delivery Method** | **Objective** | **Dates** |
| --- | --- | --- | --- |
| Project/Collaborative Introductory Webinar | Webinar | - Provide overview of program - Identify team members - Review project goals - Secure commitment from interested member organizations | January 2018 |
| Expression of Interest | Email | - Identify teams - Accept commitment - Clearly define team roles and accountability | February 2018 |
| Confirmed Participants Kick-off Webinar | Webinar | - Implement current state mapping - Gather data - Define role expectations | February 2018 |
| Touchpoint | Coaching Call | - Provide individual team orientation - Introduce DMAIC framework - Respond to any pre-conference questions | March 2018 |
| Conference | Face-to-face | - Educate on the science - Continue building out the DMAIC framework; walk through/teach improvement strategies - Create a project plan - Complete activities (poster session, panels, tours, networking) | May 2018 |
| Touchpoints | Webinar | - Build additional content based on FAQs - Address topics that meet the needs of multiple members - Focus on improvements | June - October |
| Touchpoints | Coaching Calls | - Check in on individual team progress - Focus on improvements | June - October |
| Six Month Project Report Out | Face-to-face | - Teams report out on project status - Celebration - Provide education on tactics to sustain the gains, control and dissemination - Focus on control planning | November 2018 |
| Follow-up | Teleconference | - Lessons learned & successes - Work yet to be done - Sustainment measures - Monitoring & metrics - Diffusion | June 2019 |
